# Early adversity and psychiatric symptoms – a prospective study on Ethiopian mothers and their children

**DOI:** 10.1186/s12888-017-1500-2

**Published:** 2017-10-11

**Authors:** Johan Isaksson, Negussie Deyessa, Yemane Berhane, Ulf Högberg

**Affiliations:** 1 Department of Neuroscience, Child and Adolescent Psychiatry Unit, Uppsala University, Uppsala, Sweden; 2 Department of Women’s and Children’s Health, Paediatric Neuropsychiatry Unit, Centre for Neurodevelopmental Disorders at Karolinska Institutet (KIND), Karolinska Institutet, Stockholm, Sweden; 3 Department of Preventive Medicine, School of Public Health, Addis Ababa University, Addis Ababa, Ethiopia; 4 Addis Continental Institute of Public Health, Addis Ababa, Ethiopia; 5 Department of Women’s and children’s health, Uppsala University, Uppsala, Sweden

**Keywords:** Perinatal, Maternal distress, Intimate partner violence, Programming theory, Child psychiatric symptoms

## Abstract

**Background:**

Maternal exposure to adversity during the perinatal period has been associated with increased susceptibility for psychiatric symptoms in the offspring. The aim of this study was to investigate a possible developmental effect of maternal perinatal stressors on emotional and behavioural symptoms in the offspring in a developing country.

**Methods:**

We followed an Ethiopian birth cohort (*N* = 358), assessing intimate partner violence (IPV) and maternal psychiatric symptoms during the perinatal period and at follow-up 10 years later, as a proxy for adversity, and maternal ratings on the Child Behavior Checklist (CBCL) 10 years later as the outcome.

**Results:**

Among the women, exposure to IPV was common (60.6%) during the perinatal period and predicted IPV (29.9% of the mothers) at follow-up (*ρ* = 0.132; *p* = 0.012). There was also an association between maternal psychiatric symptoms at the two time points (*ρ* = 0.136; *p* = 0.010) and between maternal symptoms and IPV. Current maternal symptoms of anxiety and depression (*β* = 0.057; *p* < 0.001), but not during the perinatal period, were associated with child CBCL-scores.

**Conclusion:**

Our findings do not support the hypothesis that early adversity increase susceptibility for psychiatric symptoms. However, the findings emphasize the public health problem of IPV in this population, adding to the women’s mental health problem.

## Background

Maternal exposure to stress or adversity during pregnancy has been associated with developmental outcomes, including preterm birth, low offspring birth weight and child death [1–4]. Lately, more long-term health outcomes of the offspring have been investigated, applying a “foetal programming theory” or a “developmental origins of health and disease hypothesis” on behavioural and emotional development of the child, including psychiatric symptoms during childhood and adolescence [3, 5–10]. Within these frameworks, early antenatal developmental influences during critical or sensitive periods of development are emphasized as possible important determinants for future mental health, and programming in this respect means an organizational effect on biological systems, such as permanent and/or epigenetic modification of gene expression, an increase sensitivity and an affected ability to react and adapt to subsequent environmental influences [10, 11].

Most research on foetal developmental programming has been made on animals and studies on humans are, for ethical reasons, mostly observational and often limited by a retrospective design. There is a need for more longitudinal studies and also those including covariates that may affect the relationship between maternal mental health in pregnancy and foetal development [3, 8]. Some prospective studies have been conducted, applying a foetal developmental programming framework, and these report of an association between maternal mood (distress, depression and anxiety) during the perinatal period and an increased risk of psychiatric symptoms in the children. For instance, maternal anxiety during pregnancy has been shown to predict child symptoms of anxiety at preadolescence in a sample of 178 mother-child pairs in the USA, independently of obstetric risk factors, socioeconomic status and current maternal wellbeing [12]; of attention problems measured at five years of age and anxiety measured up to adolescents in a study of almost 4000 pairs in Australia, adjusting for possible perinatal confounders [13]; and of behavioural and emotional problems, adjusting for psychosocial and obstetric risk, as measured with the strengths and difficulties questionnaire in children up to the age of 13 using a large English cohort of nearly 8000 pairs [14]. In addition, maternal stress during pregnancy has predicted symptoms of Attention Deficit/Hyperactivity Disorder in 7-year-olds in a Swedish sample of 290 mother-child pairs, adjusting for sociodemographic factors [15]. Studies examining intimate partner violence (IPV) and psychiatric symptoms in the offspring are scarce and are often limited to infancy and pre-school years. A recent publication, including a sample of 119 mother-child pairs in the USA, reported an association between IPV during pregnancy and internalizing and externalizing symptoms in the offspring at 10 years of age [16], even after adjusting for maternal distress and later maternal exposure to IPV. In summary, these studies support the notion that maternal psychiatric symptoms and mental disorder during the antenatal period have long-term effect on the offspring’s development, which the authors contribute to a possible programming mechanism influencing the foetal nervous system.

Despite an increasing number of publications suggesting that maternal psychiatric symptoms, such as anxiety and exposure to IPV, is an important factor to be considered in foetal development, there is a limited amount of research exploring psychiatric symptoms in children outside Northern American and European settings. Thus, there is an imperative to investigate whether these results, relating to potential early programming during the perinatal period, are valid in societies in developing countries. Within the design of a larger prospective research programme [17] following an Ethiopian birth cohort, we saw an opportunity to approach these issues using follow-up data for children at the age of ten years. The sample intends to represent young children from a poor population in a developing country and a previous study has shown that there is a high prevalence of physical and sexual IPV in Ethiopia, as compared to other countries, with 70.9% (most sites ranged between 29 and 62%) reporting life time exposure and 53.7% (most other sites ranged between 15 and 34%) reporting exposure to IPV during the last year [18]. Ethiopia was also distinguished in this study by a high percentage of women without education (84.8%). Thus, we aim to study the developmental origins to the offspring’s susceptibility to psychiatric symptoms in a low-income setting, investigating an association with maternal psychiatric symptoms and exposure to IPV during the perinatal period, while adjusting for possible confounders such as current maternal symptoms of anxiety and depression, as well as current IPV.

## Methods

### Study sample

The characteristics of the participants are presented in Table 1. This study departed from the data collection of the WHO Multi-Country study on women’s health and life events involving 15 sites in ten countries [18], where the procedure is described in more detail. The Ethiopian site included women residing in the Butajira Rural Health Program (BRHP) area 130 km south of Addis Ababa, age between 15 and 49 years, found in the list of women in the BRHP database and living in the site at least for the last three months [2, 18, 19]. Women who were still living in the demographic surveillance site of Butajira Rural Health Program, 130 km south of Addis Ababa, and had given live birth within one year (*N* = 561, out of *N* = 3016) after the baseline interview, were included in this follow-up study and were thus re-visited during 2012. After excluding women who out-migrated, women who lost the index child to death, and women who had not given birth within one year or were pregnant with the child at the timing of the interview, 358 mothers remained in the study. Data were collected through face-to-face interviews at mothers’ homes in 2002 and 2012 by trained female interviewers (women who had completed high school).

**Table 1:** Sociodemographic characteristics of the mothers at 2012 (*n* = 358)

Variable	Category	Percent
Religion %	Muslim	88.5
	Christian (all types)	11.5
Education %	Illiterate:	86.6
	≥ Elementary:	13.4
Marital status %	Married	94.7
	Divorced	1.1
	Widowed	4.2
Residency %	Rural	91.1
	Urban	8.9
Poverty status %	Poor	30.2
	Moderate	40.5
	Better off	29.3

### Questionnaires

#### Sociodemographic

The interviewers were trained using the WHO standardized women’s health and life event questionnaire (WHO, 1997) to assess such sociodemographic characteristics as age, residency and religion. Socioeconomic status (SES) was defined based on the summation of 22 items in participants’ possession, and a categorisation was created based on quartiles: absolute poverty, relatively moderate, and relatively better off. This possession summation index is based on The WHO multi-country study questionnaire and has previously been used to measure SES in Ethiopia [20].

#### Maternal psychiatric symptoms

In 2002, the Self-Reporting Questionnaire (SRQ) 20 was used as a screening instrument for non-psychotic mental disorders in the preceding 30 days. The 20 items include somatic, anxiety and depressive symptoms. Items also include questions about suicidal ideation and functional impact. The SRQ-20 has been recommended as a screening tool for common mental disorders in low- and middle-income countries [21]. In this study we used the total score, which ranges from 0 to 20. Cronbach’s alpha was in this study 0.66. In 2012, the Kessler Psychological Distress Scale (K10) was used as a screening measurement for symptoms of anxiety and depression [22]. The K10 comprises 10 items ranging from 0 = none of the time to 5 = all of the time. Cronbach’s alpha for the scale was in our study 0.85.

#### Intimate partner violence

The WHO standardized questionnaire and categories [18] was used to assess severe (physical or/and sexual) IPV during the last year. Six questions assessed exposure to physical violence: “Has the partner slapped or thrown items at the women”, “pushed or shoved”, “being hit”, “being kicked, dragged or beaten”, “choked or burned”, and “threatened with weapon/knife”? Three questions assessed sexual violence: “Physically forced to have sex”, “forced to have sex that made her afraid”, and “degrading sex”? IPV was scored as: 0 for no exposure to physical or sexual violence during the last year, 1 for exposure to either physical or sexual violence, and 2 for exposure to both physical and sexual violence during the last year.

#### Child emotional and behavioural symptoms

The women were interviewed with the Child Behavior Check-list (CBCL/6–18) [23], which has 120 statements about the child on a 3-point scale (0 for not true, 1 for somewhat or sometimes true, and 2 for very true or often true). The points were summarized and transferred to a total problem score, and total internalizing and externalizing symptom scores. Internalizing symptoms refer to symptoms directed inward, as in depression, anxiety, and somatic complaints; whereas externalizing symptoms are displayed outwardly as in disruptive behavior disorders [24]. Cronbach’s alpha for the total problem scale in this study was 0.93.

### Statistical analyses

All statistical analyses were performed using the Statistical Package for Social Sciences (IBM SPSS version 22). Because the CBCL-scores were skewed (a skewness ratio above 1.96), correlations were calculated using Spearman’s rho and we used a generalized linear model with a gamma distribution and log link in the regressions models. We performed three multifactorial models with the CBCL total score, internalizing score and externalizing score, respectively, as dependent variables; as independent variables we used ratings on maternal psychiatric symptoms, exposure to IPV, SES, and the child’s sex. Finally, as a post-hoc analysis, we tested if exposure to IPV at both time points, compared to one or no exposure, was associated with different CBCL ratings. Two-tailed tests with *p* values < .05 were considered significant.

## Results

The majority (76%, *N* = 272) of mothers had given birth to the child within the year before the interview, and 24% (*N* = 86) were pregnant during the interview. In 2012, the mean age of the offspring (*N* = 358; females *N* = 159 [44.7%]; males: *N* = 197 [55.3%]; not specified: *N* = 2) was 10.3 years (SD = 0.43). Among the women, the mean value for the SRQ-20 at baseline was 2.47 (SD = 2.56), and 4.48 (SD = 4.57) for the K10 ten years later. When applying commonly used cut-off points (≥6 points on SRQ-20 and ≥10 points on K10), 12.8% at baseline and 12.6% ten years later were likely to have at least a minor mental disorder. Regarding IPV, 60.6% (*N* = 217) of the women had been exposed to severe partner violence during the last year (22.9% had been exposed to both physical and sexual violence) at baseline. At follow-up, 10 years later, the same number was 29.9% (*N* = 107, of which 5% had been exposed to both physical and sexual violence), and 20.9% of the women were exposed to IPV both 2002 and 2012. There was a positive correlation between maternal psychiatric symptoms for 2002 and 2012 (*ρ* = 0.136; *p* = 0.010) and maternal exposure to IPV (*ρ* = 0.132; *p* = 0.012) at the two time points (Table 2). Psychiatric symptoms in the mothers were positively correlated with IPV, rated in both 2002 (*ρ* = 0.105, *p* = 0.047) and 2012 (*ρ* = 0.151, *p* = 0.004). CBCL mean scores for the children were: for the total score 13.52 (SD = 13.56), for internalizing symptoms 5.27 (SD = 5.35), and for externalizing symptoms 3.06 (SD = 3.72). A minority of the children were rated above a clinical cut-off (for internalizing symptoms, 12% of the boys and 13% of the girls; for externalizing symptoms, 4% of the boys and 2.5% of the girls, respectively).

**Table 2:** Correlations (Spearman’s rho) between data collected during the perinatal period, at follow-up, and maternal ratings of child psychiatric symptoms

	Maternal SRQ-20 scores (2001)	Maternal IPV (2001)	SES (2001)	Maternal K10 scores (2012)	Maternal IPV (2012)	SES (2012)	Child sex	Child Total CBCL	Child internalizing symptoms	Child externalizing symptoms
Maternal SRQ-20 scores (2001)	*ρ* = 1.00									
Maternal IPV (2001)	*ρ* = 0.105*	*ρ* = 1.00								
SES (2001)	*ρ* = −0.027	*ρ* = −0.015	*ρ* = 1.00							
Maternal K10 scores (2012)	*ρ* = 0.136*	*ρ* = 0.010	*ρ* = −0.011	*ρ* = 1.00						
Maternal IPV (2012)	*ρ* = 0.071	*ρ* = 0.132*	*ρ* = −0.004	*ρ* = 0.151**	*ρ* = 1.00					
SES (2012)	*ρ* = −0.002	*ρ* = 0.012	*ρ* = 0.111*	*ρ* = −0.191***	*ρ* = 0.015	*ρ* = 1.00				
Child sex (male)	*ρ* = 0.017	*ρ* = –0.013	*ρ* = 0.008	*ρ* = –0.038	*ρ* = 0.063	*ρ* = 0.016	*ρ* = 1.00			
Child Total CBCL	*ρ* = 0.030	*ρ* = −0.064	*ρ* = 0.037	*ρ* = 0.190***	*ρ* = 0.069	*ρ* = −0.099	*ρ* = 0.075	*ρ* = 1.00		
Child internalizing symptoms CBCL	*ρ* = 0.005	*ρ* = −0.016	*ρ* = 0.028	*ρ* = 0.190***	*ρ* = 0.089	*ρ* = −0.120*	*ρ* = 0.013	*ρ* = 0.918***	*ρ* = 1.00	
Child externalizing symptoms CBCL	*ρ* = 0.031	*ρ* = −0.084	*ρ* = 0.080	*ρ* = 0.145**	*ρ* = 0.097	*ρ* = −0.015	*ρ* = 0.121*	*ρ* = 0.889***	*ρ* = 0.732***	*ρ* = 1.00

Significance: *p < 0.05, **p < 0.01, ***p < 0.001; CBCL = Child Behavior Check-list; IPV = Intimate partner violence; K10 = The Kessler Psychological Distress Scale; SRQ-20 = Self-Reporting Questionnaire

In the multivariable GLMs, current maternal psychiatric symptoms were associated with the rated total problems (*β* = 0.057; *p* < 0.001; CI = 0.036–0.078; OR = 1.059), internalizing symptoms (*β* = 0.057; *p* < 0.001; CI = 0.036–0.078) and externalizing symptoms (*β* = 0.055; *p* < 0.001; CI = 0.034–0.077: OR = 1.057) in the child (Table 3). Also, boys had higher rated externalizing symptoms in the model (*β* = 0.342; *p* = 0.001; CI = 0.157–0.527; OR = 1.408). None of the maternal psychiatric symptoms at baseline (*β* = 0.006; CI = −0.030–0.041; OR = 1.006), or IPV in 2002 (*β* = −0.089; CI = −0.207–0.030; OR = 0.915) and 2012 (*β* = −0.008; CI = −0.172–0.155; OR = 0.922), or SES (*β* = 0.047; CI = −0.098–0.193; OR = 1.048 and *β* = 0.026; CI = −0.097–0.149; OR = 1.026, respectively) were associated with child total CBCL ratings. No interaction effects were found between maternal psychiatric symptoms and IPV, or between sex and the covariates, on CBCL ratings.

**Table 3:** Generalized linear model of the association between data during the perinatal period and 10-years-later on child psychiatric symptoms (total score)

Predictors	*β* (CI)
Maternal psychiatric symptoms (SRQ-20) 2002	0.006 (−0.030–0.041)
(K10) 2012	0.057 (0.036–0.078)* * *
Exposure to IPV 2002	−0.089 (−0.207–0.030)
2012	−0.008 (−0.172–0.155)
Sex (male)	0.136 (−0.044–0.315)
SES 2002	0.047 (−0.098–0.193)
2012	0.026 (−0.097–0.149)

Significance: ***p < 0.001; CBCL = Child Behavior Check-list; IPV = Intimate partner violence; K10 = The Kessler Psychological Distress Scale; SRQ-20 = Self-Reporting Questionnaire

In the post-hoc analysis, investigating any possible effect of chronic exposure to IPV, no difference in CBCL ratings was found between mothers never exposed to IPV, only at one time point or in both 2001 and 2012.

## Discussion

In this prospective study on a sample of Ethiopian mothers and their children, neither maternal psychiatric symptoms nor exposure to intimate partner violence (IPV) during the perinatal period predicted child emotional and behavioural symptoms when the offspring was 10 years old. Current maternal symptoms of depression and anxiety during follow-up were, however, associated with rated emotional and behavioural symptoms in the child. IPV was common during the perinatal period and also predicted exposure to IPV at follow-up, even though the prevalence had decreased by half. There was a weak correlation between IPV and maternal psychiatric symptoms.

Previous research on the effect of stress or adversity during pregnancy on developmental outcomes has primarily focused on perinatal data such as preterm birth and low offspring birth weight. Studies made on animals have provided support to the hypothesis of foetal developmental programming on more long-term behavioural and emotional outcomes [3, 5–10]. There have also been human studies, presenting an association between early adversity such as maternal anxiety or IPV and psychiatric symptoms during childhood [12–16]. However, in our sample from a developing country, no association was found, thereby not giving support to the hypothesis of an early developmental effect of perinatal stress on behaviour and emotional symptoms in the offspring. This negative finding is in line with previous research from our research group, completed with a Nicaraguan sample of women and school-aged children [25] and in a recent review on impact of maternal antenatal depression on the offspring’s temperament, cognitive and emotional development, it was concluded that most studies find either no impact or only a small effect, with the notable exception of conduct problems and antisocial behaviour in the child [26].

Our finding that current maternal symptoms of anxiety and depression were associated with rated psychiatric symptoms of the child, rather point towards psychosocial explanations, possibly corresponding with deficiency in nurturing or stimulating qualities of the caregiving environment, factors that may affect mental health problems in children [27]. However, even though no support was found for any foetal developmental programming, it should not be concluded that IPV during the antenatal period have no effect on the offspring development. Possibly, other developmental outcomes, not captured with CBCL ratings, may be affected. Also, because IPV was so common in our population, reports on IPV may not be a relevant measure of significant stress within this population.

More than half (60.6%) of the mothers had been exposed to severe IPV during pregnancy, which is in line with a previous study, comparing the prevalence of physical and sexual IPV in 10 countries, where Ethiopia had the highest reports with 53.7% of women having reported physical or sexual violence within the previous 12 months [18]. The authors argue that IPV may be higher in more traditional and rural environments with a low empowerment of women. As a contrast, maternal psychiatric symptoms were not elevated, even though there was a weak correlation between IPV and psychiatric symptoms. At baseline, 12.8% of the women were likely to have a disorder, and 12.6% ten years later, and, in a review on the prevalence of non-psychotic common perinatal mental disorders in low- and lower-middle-income countries, the mean prevalence was 15.6% during pregnancy and 19.8% postnatally [28].

There are several limitations to this study. First, several factors not included in this study may affect psychiatric symptoms, for instance, paternal psychiatric symptoms, birth weight, and traumas, but also, positive influences, such as resources and school characteristics, were also omitted. Thus, no causal associations can be assessed. Also, we lack genetic data and an optional design would be to include data on siblings not exposed, thereby adjusting for numerous possible confounders. It would also be of interest to include data from biological systems that may mediate any possible programming effect, such as the hypothalamic-pituitary-adrenal axis, the immune system and the gut microbiome [11]. Second, data on child emotional and behavioural symptoms were only collected from the mothers, meaning that we only have one perspective of the child’s mental status. Maternal reports may be biased by their own wellbeing and the mothers may have had difficulties in differentiating between types of problems. To focus on psychiatric diagnoses it would be preferable to use data from other information sources and from structured diagnostic interviews as well. Third, the timings of exposure to IPV and psychiatric symptoms were not specified, which means that exposure may have occurred at several time points, during different trimesters, or even slightly before or after pregnancy. However, we regard exposure to IPV or symptoms such as anxiety and depression as a proxy for adversity during the perinatal period and the exposure to IPV is probably not an expression for any one single occasion. Forth, the questionnaire on maternal psychiatric symptoms differs between the two time-points. However, SRQ-20 and Kessler are comparable instruments in sensitivity and specificity to detect major depressive disorders [29]. The possible strengths of this study were the longitudinal design in an under-studied population and the inclusion of socio-economic status and current adversity, which may be a possible confounder. For future studies, the assessment of early adversities may be supplemented with biomarkers such as cortisol, and might also including a more structured assessment of the children’s cognitive and behavioural development over the years.

We conclude that our main findings do not support the hypothesis of an early developmental effect of perinatal adversity with increased susceptibility for psychiatric symptoms. Rather, rated child emotional and behavioural symptoms corresponded with maternal mental health, stressing the need for intervention promoting maternal health within this population. Sadly, exposure to severe IPV was common within this population of mothers and correlated with both maternal psychiatric symptoms and IPV ten years later, and one fifth of the women had been exposed at both time-points. These findings emphasize the public health problem of IPV, adding to the burden of women’s mental health suffering and difficulties in child rearing affecting also the children’s mental health. The high frequency of IPV needs to be targeted with interventions suitable for this population.

## Abbreviations

CBCL: Child Behavior Checklist
IPV: intimate partner violence
K10: Kessler Psychological Distress Scale
SES: Socioeconomic status
SRQ: Self-Reporting Questionnaire
